# Genetic Spatiotemporal Anatomy of *Plasmodium vivax* Malaria Episodes in Greece, 2009–2013

**DOI:** 10.3201/eid2403.170605

**Published:** 2018-03

**Authors:** Gregory Spanakos, Georges Snounou, Danai Pervanidou, Michael Alifrangis, Anna Rosanas-Urgell, Agoritsa Baka, Maria Tseroni, Annita Vakali, Evdokia Vassalou, Eleni Patsoula, Herve Zeller, Wim Van Bortel, Christos Hadjichristodoulou, for the MALWEST Project

**Affiliations:** Hellenic Center for Disease Control and Prevention, Marousi, Greece (G. Spanakos, D. Pervanidou, A. Baka, M. Tseroni, A. Vakali);; Sorbonne Universités, Paris, France (G. Snounou);; Institut National de la Santé et de la Recherche Médicale, Paris (G. Snounou);; ERL Centre National de la Recherche Scientifique, Paris (G. Snounou);; University of Copenhagen, Copenhagen, Denmark (M. Alifrangis);; Institute of Tropical Medicine, Antwerp, Belgium (A. Rosanas-Urgell);; National School of Public Health, Athens, Greece (E. Vassalou, E. Patsoula);; European Centre for Disease Prevention and Control, Stockholm, Sweden (H. Zeller, W. Van Bortel);; University of Thessaly, Larissa, Greece (C. Hadjichristodoulou)

**Keywords:** malaria, Greece, genetic spatiotemporal anatomy, Plasmodium vivax, parasites, migrants, transmission, microsatellite marker, PvMSP-3α

## Abstract

An influx of immigrants is contributing to the reemergence of *Plasmodium vivax* malaria in Greece; 1 persistent focus of transmission is in Laconia, Pelopónnese. We genotyped archived blood samples from a substantial proportion of malaria cases recorded in Greece in 2009–2013 using 8 microsatellite markers and a *PvMSP-3α* gene fragment and plotted their spatiotemporal distribution. High parasite genetic diversity with low multiplicity of infection was observed. A subset of genetically identical/related parasites was restricted to 3 areas in migrants and Greek residents, with some persisting over 2 consecutive transmission periods. We identified 2 hitherto unsuspected additional foci of local transmission: Kardhítsa and Attica. Furthermore, this analysis indicates that several cases in migrants initially classified as imported malaria were actually locally acquired. This study shows the potential for *P. vivax* to reestablish transmission and counsels public health authorities about the need for vigilance to achieve or maintain sustainable malaria elimination.

The global strategies for malaria control and elimination have led to substantial decreases in malaria incidence worldwide (*1*). In countries outside of Africa, *Plasmodium vivax* often predominates, making this species the most widespread malaria parasite. A combination of *P. vivax* biologic characteristics hamper its elimination and facilitate its reintroduction. The hypnozoite (i.e., the parasite during the dormant liver stage) can activate after months and cause new episodes, extending the period of carriage and transmission. The wide range of anopheline vectors that are susceptible to *P. vivax* and the capability of sporozoites to mature in lower temperatures extends this parasite’s geographic range. Also, compared with *P. falciparum*, *P. vivax* infections have low peak parasitemias and clinical immunity (protection against clinical manifestations) is rapidly acquired, resulting in infections that are often asymptomatic, which can prevent prompt diagnosis and treatment and prolong the duration of infection (*2*).

Episodes of rapid resurgence of *P. vivax* malaria have occurred as a consequence of substantial disruption of public health services and large-scale population displacement triggered by war, civil unrest, or socioeconomic crises (*3*,*4*). In addition, with the increase in international travel and migration, sporadic cases of autochthonous infections due to transmission from carriers are noted yearly in various nonendemic countries (*5*). In South Korea (declared malaria-free in 1979), reintroduction of *P. vivax* malaria followed by sustained transmission was observed. Starting with a few cases recorded in the early 1990s in military personnel posted at the demilitarized zone that bisects the Korean Peninsula, the numbers increased to a peak of ≈4,100 by year 2000, with soldiers, veterans, and civilians nearby the demilitarized zone equally affected (*6*). Infected mosquitoes from North Korea were responsible for the introduction of *P. vivax* and probably still contribute to transmission (*7*).

During 2009–2012, episodes of reintroduced autochthonous *P. vivax* malaria occurred in Greece, the first such episodes in Europe since the 1970s. Greece was hyperendemic for malaria before an intense malaria eradication program (in 1946–1960), which led to the country becoming malaria-free in 1974, with a small number of imported malaria cases recorded annually thereafter (*8*). A threat to Greece’s malaria-free status arose with a major upsurge in the number of undocumented migrants from *P. vivax*–endemic countries, principally the Indian subcontinent (estimated 200,000 migrants during 2006–2012) (*9*). During 2009–2013, nearly one fourth (27/113) of migrants with imported cases of *P. vivax* malaria reported a history of malaria in their country of origin (C. Hadjichristodoulou, unpub. data). The Hellenic Center for Disease Control and Prevention (HCDCP; Athens, Greece) declared these 113 cases as imported malaria (defined as occurring in persons arriving from endemic areas). HCDCP also defined *P. vivax* cases occurring in migrants <3 years after their arrival to Greece as relapses (recrudescence from persistent subpatent blood infections [i.e., *P. vivax* infections in which the pathogen is present but not detectable by standard laboratory methods] was considered unlikely, although it could not be formally excluded). The potential for local transmission increased substantially in receptive rural areas where migrants settled to serve as seasonal agricultural workers, thereby bringing carriers in contact with the local anopheline vectors during the transmission season. Of note, vector control activities after 1974 (after Greece was declared malaria-free) were not focused on *Anopheles* mosquitoes but rather on those that were considered a nuisance to the local population.

In 2009, an initial cluster of *P. vivax* malaria in 6 Greece residents (defined here as persons living in Greece with no travel history to *P. vivax* malaria–endemic areas) in a small agricultural locality in southern Pelopónnese (regional unit Laconia, municipality Evrótas) alerted the HCDCP. Only 1 such locally acquired (autochthonous) case was reported in Evrótas in 2010 (*10*,*11*), but in 2011, a further 36 locally acquired cases in Greece residents and 21 cases classified as imported in migrant workers were recorded in the same area; these 57 cases represented ≈60% of all cases recorded in Greece that year. Despite prompt deployment of classic malaria prevention measures (mosquito control, distribution of insecticide-treated bed nets, fever screening of immigrants, and investigation of each malaria case) during the 2011 and 2012 transmission seasons, 27 more cases (17 imported in migrants and 10 in Greece residents) occurred in Evrótas in 2012. The next year, the same measures were reinforced by the deployment of mass drug administration targeting the migrant workers in the area (*12*), a strategy that seemed to have contributed to successfully thwarting the reestablishment of the parasite, considering that no autochthonous cases were recorded from this regional unit in 2013 and 2014. During the same period (2009–2013), 74 imported *P. falciparum* cases were recorded in Greece, but none were in Laconia.

In another area, a village in regional unit Kardhítsa, Thessalía, 11 *P. vivax* malaria cases (2 locally acquired in Greek residents and 9 in migrants) were recorded in 2012. In addition, 1 locally acquired case was recorded in 2013.

The availability of archived blood samples from a substantial proportion of all the *P. vivax* malaria cases recorded in Greece during 2009–2013, including those from Laconia, provided the opportunity to investigate in detail the spatiotemporal distribution of *P. vivax* introduction during this period through a molecular description of select parasite polymorphic genetic markers (*13*). Here we report on the spatiotemporal genotypic data from these cases and discuss the insights the data provide on the potential for *P. vivax* to establish sustained transmission in vulnerable receptive areas.

## Materials and Methods

### *P. vivax* Blood Samples

**Figure 1:**
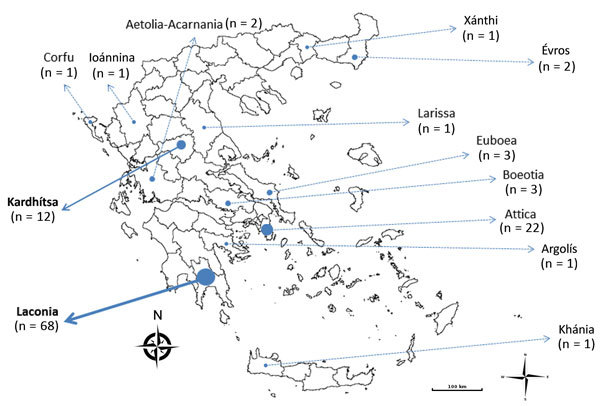
Geographic origin of *Plasmodium vivax* cases analyzed, Greece, 2009–2013. The 2 foci of transmission are Laconia and Kardhítsa (in bold). Size of dots is proportional to number of cases. Samples from Attica were distributed widely throughout this large regional unit, which includes Athens.

During 2009–2013, a total of 311 malaria cases were reported to the HCDCP, of which 209 were caused by *P. vivax* as confirmed by microscopy or PCR analysis. All cases were symptomatic and recorded either through the passive surveillance system (hospitals, health centers) or through active surveillance activities, which included focus investigations of autochthonous cases and fever screening among immigrants in Laconia (*12*). Of the 209 recorded *P. vivax* cases, 122 blood samples collected from 118 patients were available for genotyping (Figure 1). Of the 53 Greece residents, 4 were Romanian, 4 were Moroccan, and 1 was Polish. The study protocol was approved with a waiver of informed consent by the Scientific Committee of the Postgraduate Program of Applied Public Health and Environmental Hygiene, Faculty of Medicine, University of Thessaly, Larissa, Greece.

### Genomic DNA Isolation

We isolated genomic DNA from blood samples using the QIAamp DNA Mini Kit (QIAGEN GmbH, Hilden, Germany), the Magcore automated system (RBC Bioscience, New Taipei City, Taiwan), or iPrep PureLink gDNA Blood Kit (Invitrogen, Carlsbad, CA, USA). The last method was used exclusively for the samples collected late in 2012 and in 2013.

### Genotyping Analysis

We subjected *P. vivax* populations to genotyping targeting 9 main polymorphic markers: 8 microsatellite loci and a region of the gene encoding *PvMSP-3α*. We pooled data available from a previous study for a subset of the samples (genotyped for a subset of markers) with the data we obtained using additional samples and microsatellite markers (*13*). We performed genotyping for *PvMSP-3α* and microsatellite loci m1501 and m3502 as previously described (*13*) and for microsatellite loci MS1, MS5, MS7, MS8, MS12, and MS20 as previously described (*14*). For a subset of samples (n = 54) that yielded similar or closely related haplotypes, we performed genotyping for 4 additional polymorphic markers (3 microsatellite loci MS10, MS16, and Pv3.27, and the polymorphic F3 domain of *PvMSP1*), which are hereafter referred to as additional markers, as previously described (*15*). For all microsatellite markers, we analyzed PCR products by capillary electrophoresis using 500 LIZ or 1200 LIZ (Applied Biosystems, Foster City, CA, USA) as size standards. We used GeneMarker 2.6.0 (https://www.softgenetics.com/GeneMarker_4.php) for allele calling and considered peaks <500 relative fluorescence units noise. We checked samples manually to confirm true alleles.

We excluded samples from further analysis if <7 of the initial 9 genetic markers did not yield valid results. We classified a sample as having multiple genotype infections if >1 allelic variant with a peak height of at least a third of the main allelic variant peak was observed or, for *PvMSP-3α*, if >1 DNA band was visible in the PCR products. We considered 2 haplotypes the same only when all 9 markers shared the same allelic variant. In cases of mixed genotype infections, the dominant allelic variant for each marker was alone considered to determine the haplotype. We used the term family to denote parasites that shared >7 out of the 9 main markers. We constructed the phylogenetic tree using Populations 1.2.31 (http://bioinformatics.org/populations/) using the shared allele distance and UPGMA algorithm and visualized with Dendroscope 3 (*16*).

## Results

Of the 122 samples, we genotyped 104 successfully; 14 had 1 or 2 of the 9 markers fail to amplify, and 4 were excluded because <7 markers amplified. These 118 samples were obtained from 114 separate patients (4 patients had *P. vivax* infection and were tested in 2011 and 2012). This set comprised a substantial proportion of all the *P. vivax* cases recorded during 2009–2013 (Table). The samples were divided into 3 subsets by their geographic location (Figure 1): the first 2, Laconia (with 68 samples) and Kardhítsa (with 12 samples), were foci of transmission where multiple cases were recorded over time in a relatively restricted area, and the third encompassed the samples from the rest of Greece (with 38 samples).

**Table:** Reported *Plasmodium vivax* malaria cases, Greece, 2009–2013

Year of onset	Laconia					Kardhítsa and rest of Greece			
	Greece resident		Migrant*			Greece resident		Migrant	
	Cases	Samples	Cases	Samples		Cases	Samples	Cases	Samples
2009	6	4	2	0		1	0	11	0
2010	1	0	1	1		3	0	23	0
2011	34	19	23	11		6	5	14	3
2012†	15‡	15	18	17		10	9	39	29
2013	0	0	1	1		4§	3	8	1

*Population includes 6 persons with a travel history to endemic countries. †Includes 5 relapse cases and samples: 3 Greece residents and 1 migrant in Laconia and 1 Greece resident in the rest of Greece. ‡Includes 2 locally acquired cases attributed to the 2011 transmission period. §Includes 1 locally acquired case attributed to the 2012 transmission period.

**Figure 2:**
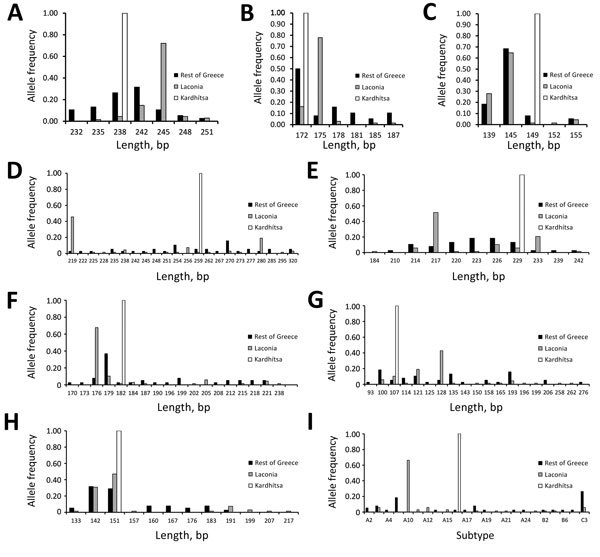
Frequency distribution of *Plasmodium vivax* allelic variants and lengths or subtype of genetic markers, by geographic location, Greece, 2009–2013. The frequencies for microsatellite markers MS1 (A), MS5 (B), MS7 (C), MS8 (D), MS12 (E), MS20 (F), m1501 (G), and m3502 (H) and gene fragment msp3 (I) are calculated separately for the samples from each of the 3 geographic sets: Laconia (n = 68), Kardhítsa (n = 12), and the rest of Greece (n = 38).

The extent of allelic diversity observed differed for the main genetic markers (range 5–23 distinct variants/marker; Technical Appendix 1 Tables 1–3), and the corresponding allelic frequency distributions differed by collection site (Figure 2, panels A–I). Mixed clone infections were detected in 18 samples, in which only 2 allelic variants were observed; among these 36 variants, 27 were the same 2 variants (12 of 1 variant and 15 of the other). Thus, most *P. vivax* cases diagnosed in Greece appeared to harbor monoclonal infections.

**Figure 3:**
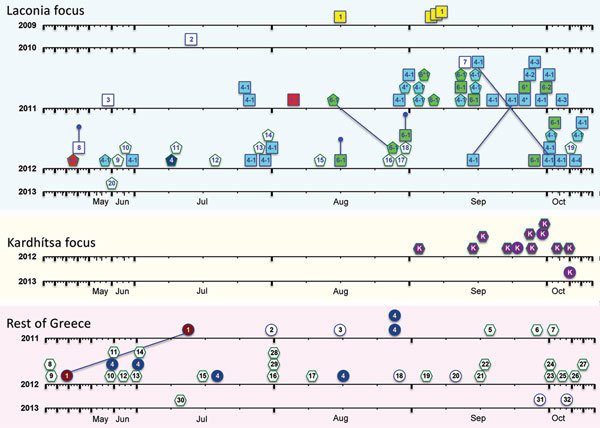
Temporal representation of the occurrence of *Plasmodium vivax* haplotypes, Greece, 2009–2013. Data for each region (Laconia, Kardhítsa, and rest of Greece) are depicted. Samples are indicated by shapes: in Laconia, squares indicate Greece residents and pentagons migrants; in Kardhítsa and rest of Greece, circles indicate Greece residents and hexagons migrants. Colors indicate haplotype families: La-1, yellow; La-4, blue; La-5, red; La-6, green; Gr-1, maroon; Gr-4, dark blue; and Ka, purple. Haplotype numbers are given inside shapes. The *x*-axis time scale for each year was stretched to accommodate the higher number of cases reported during the transmission season, with January, February, March, April, November, and December compressed. Lines joining 2 samples indicate isolates collected from the same person. Truncated vertical lines indicate the 3 samples collected in 2012 from persons considered exposed in the previous year: 1 relapse of a 2011 case and 2 patients with symptom onset in 2012 whose exposure was attributed to the 2011 transmission period. Dates of symptom onset of 2 patients tested in 2012 (green hexagons, haplotypes 28 and 29) were unknown and were arbitrarily assigned date August 1. Further details of samples are provided in online Technical Appendix 1 Tables 1–3 (https://wwwnc.cdc.gov/EID/article/24/3/17-0605-Techapp1.xlsx).

A total of 67 distinct haplotypes were observed when all 9 genetic markers were considered (Technical Appendix 1 Table 1), of which 53 were unequivocal and 14 corresponded to samples in which 1 or 2 of the markers failed to amplify. Of the 68 samples from the Laconia outbreak, we observed 28 unique *P. vivax* haplotypes only once, and we detected only 1 of these haplotypes (family Greece 4 [Gr4]) in samples collected from another region of Greece. Fourteen of the haplotypes from Laconia (observed in 51 of 68 samples) fell into 4 families: 1) family Laconia 1 (La1), which was observed in 4 samples collected in 2009; 2) La4, which had 8 subtypes and was observed in 32 samples (24 of which were subtype La4–2); 3) La5, which had 2 subtypes, each observed only once; and 4) La6, which had 3 subtypes observed in 13 samples (11 of which were subtype La6–1). One fourth (17/68) of the samples from Laconia had unique haplotypes that did not belong to these 4 haplotype families. Of the 35 haplotypes recorded outside Laconia, 1 haplotype of the family Kardhítsa (Ka) was observed solely in the 12 samples (from 3 Greece residents and 9 migrants) in regional unit Kardhítsa. Two related haplotypes (of family Gr1) were observed in the same person who had a relapse in February 2012 seven months after the first episode, and another 2 haplotypes (Gr31 and Gr32, also related) were found in 2 Greece residents from regional unit Évros who had symptoms 1 month apart from each other. Finally, 2 haplotypes of the Gr4 family were observed in 6 persons in the east of regional unit Attica (5 Greece residents and 1 migrant). The remaining 28 haplotypes were each observed only once in samples obtained throughout the rest of Greece (Figure 3; Technical Appendix 2 Figures 1, 2).

To obtain a dynamic picture of the *P. vivax* isolates that were circulating in Greece throughout the episodes of reintroduction, we displayed the haplotypes in their order of temporal appearance (Figure 3). The dispersal pattern of *P. vivax* parasites, in particular those of the major haplotype families (Gr4 in the eastern part of Attica; Ka in Kardhítsa; and La1, La4, La5, and La6 in Laconia), could be discerned. The genetic analysis of the parasites from Laconia, the main focus of reintroduced *P. vivax*, showed that the parasite haplotype found in Greek residents in 2011 and 2012, when most cases occurred, represented a restricted subset of the haplotypes found in the migrants. All but 3 of the samples acquired from 31 Greek residents (34 samples) belonged to 3 haplotype families: La4, La5, and La6. Fifteen of the 27 samples acquired from migrants during the same years also belonged to families La4–La6, with the remaining 12 samples from migrants each harboring a unique but unrelated parasite haplotype.

In Attica, 6 of the cases recorded during August 2011 and summer 2012 among Greek residents from 4 different locations were the same haplotype (Gr4); thus, Attica can be considered an additional focus of local transmission. We observed the same haplotype in an Afghani migrant who had *P. vivax* in early July 2012 in Laconia. Finally, in regional unit Kardhítsa, Thessalía, all 12 *P. vivax* samples (11 from 2012 and 1 from 2013) collected from a single village (6 of which were from a focus investigation) shared the same haplotype. Eleven of these samples were from patients of different nationalities (7 Afghani, 2 Bangladeshi, and 2 Romanian) who lived in close proximity and sought treatment within 2 months of each other.

Given that the differences between the haplotypes within each of these 6 families were minor, the corresponding samples were subjected to genotyping with 4 additional genetic markers: 3 microsatellite loci MS10, MS16, and Pv3.27, and the polymorphic F3 domain of *PvMSP1*. This analysis revealed that the Gr4 family comprises 4 subtypes that differ with respect to 1 or 2 of the additional markers. We were able to distinguish 4 subtypes of haplotype family Ka and 3 subtypes of haplotype family La4 with 1 of these 4 additional loci. The members of the La6 family were homogeneous at all these loci (Technical Appendix 2 Figures 1, 2).

## Discussion

Using archived blood samples, we determined the genetic anatomy of many of the *P. vivax* isolates introduced into Greece during 2009–2013 and investigated the dynamics of the transition of a location from a source of imported cases to an area with established, sustained autochthonous transmission. These transitions are increasingly likely to occur in areas where malaria control efforts have eliminated or will eliminate the parasite. This fact is of particular concern for *P. vivax*, whose ability to form hypnozoites substantially enhances its potential to transmit over extended periods. Standard approaches of screening and treatment are inapplicable for *P. vivax* because screening for hypnozoite carriage is not possible. Moreover, the toxicity of primaquine phosphate, the only hypnozoitocidal drug available, precludes its use without prior onerous testing for glucose-6-phosphate dehydrogenase deficiency. Thus, the often-asymptomatic or clinically mild relapses that might occur in migrants over time provide additional opportunities for local anopheline mosquitoes to transmit the parasite locally.

A total of 1,320 cases of malaria were reported in Greece during 1975–2008, a mean of 39 (range 16–79) cases/year; 475 of these cases were *P. vivax* diagnosed in visitors from the Indian subcontinent. Over this period only 13 autochthonous cases (2 in 1975, 3 in 1976, 5 in 1991, 1 in 1999, and 2 in 2000) occurred in diverse regions of Greece (*8*). In contrast, during 2009–2013, *P. vivax* malaria was diagnosed in 139 migrants or travelers from endemic countries and 76 Greece residents (Table). Most likely, the substantial increase after 2006 in numbers of Indian subcontinent migrants, with many employed over the spring and summer months as agricultural workers, led to the reintroduction of *P. vivax* in some rural areas. The focus of introduced *P. vivax* malaria was considered to be in regional unit Laconia because 53 of the 76 cases in Greece residents were from 6 villages in Laconia (municipality Evrótas) within a 2.5-km radius, with the remaining 23 being from diverse and distant regions of Greece. The identification of the same haplotypes as in an earlier study, in which a combination of only 3 markers were used to analyze samples from 2 consecutive years (2011 and 2012), suggested that sustained transmission in the area was possible and further measures were needed to control the disease. Thus, mass drug administration with the immigrant population in Laconia was decided on and performed as a supplementary measure (*12*).

Our detailed spatiotemporal genetic analysis, in which we identified and compared the *P. vivax* haplotypes in patient samples, helped shed light on the epidemiology of reintroduced *P. vivax* episodes. Some of the infections in migrants in Laconia in 2011–2012 were most probably acquired locally and should consequently be reclassified as autochthonous. Careful analysis of the genotyping data uncovered 2 additional foci of local transmission and confirmed the need to reevaluate the classification of cases as autochthonous and imported. Despite the high number of cases, the focus in Attica was unsuspected because cases were scattered throughout the region. Genotyping revealed that parasites from 6 unrelated Greece residents (8 samples, 2 in 2011 and 6 in 2012) living within a 15-km radius of each other shared the same haplotype (Gr4). This haplotype was also found in 2012 in 1 migrant living in Laconia, but further investigation did not reveal any epidemiologic connection between this case and those from Attica.

The cluster of 11 cases recorded in 2011 in Kardhítsa was not thought of as a focus of local transmission because 9 of these cases were in migrants from the Indian subcontinent. However, genotyping revealed that the parasites from all 11 migrants were of the same haplotype, as were those from a Greece resident with no history of malaria or travel in 2013. This infection might have been acquired during the previous months from an asymptomatic carrier, or the infection might represent a delayed primary attack after inoculation by an infected mosquito the previous year. Thus, Kardhítsa represents a third focus of local transmission, where many of the migrant cases classified as imported malaria by using classic epidemiologic criteria should be reclassified as locally acquired.

From an epidemiologic point of view, *P. vivax* parasites have a high potential for initiating and maintaining local transmission within a short period of time from carriers reaching receptive areas hitherto free of malaria. In such areas, the nonimmune status of the local population will facilitate rapid detection of cases, considering that infected, previously malaria-naive residents would generally have clinical symptoms that are sometimes severe. However, in regions where *P. vivax* malaria has been eliminated or brought to very low incidence, the imported parasite strains might persist as subpatent infections because of residual levels of acquired immunity in the local population, which would enhance the likelihood of wider dissemination. Nonetheless, data from the reintroduction episodes we present suggest that the potential for establishing transmission might differ by parasite strain, considering that relatively few *P. vivax* strains that were introduced into Greece during the 5 years of our analysis led to clusters of sustained local transmission. The difference in transmission might be attributable to biologic or environmental factors or a combination of both. The local mosquito populations might be inefficient at transmitting some of the imported strains (*17*), and variation in the vector midgut bacterial biota might also restrict transmission (*18*). However, the 3 foci we identified involved genetically diverse parasites (Technical Appendix Figure 2), which could be explained by the parasites having differential transmission efficacies for the local anopheles populations. Finally, the degree of human–anopheline contact also plays a crucial role, such that the parasites in carriers residing in a rural area with a high mosquito density are more likely to be transmitted than parasites in carriers living in an urban environment with a low mosquito density.

The reintroduction of *P. vivax* malaria into Greece during 2009–2013 should serve as a warning to those engaged in efforts to eliminate malaria (*19*) in countries where *P. vivax* predominates, especially for those in countries close to elimination and in malaria-free areas neighboring *P. vivax*–endemic regions. The prompt and flexible countermeasures brought to bear by the Greek public health infrastructure were effective in containing and then extinguishing the foci of transmission (*12*). In our genotypic analyses, we highlight 2 epidemiologic features: first, the high potential for imported *P. vivax* to establish sustainable transmission within a short time; and second, the difficulty in predicting the locations where such transmission might occur. *P. vivax* is a recognized obstacle for malaria elimination (*20*). Maintaining vigilance will be crucial to ensure that this parasite will not threaten those countries that achieve it.

Technical Appendix 1 Haplotype description of 118 *Plasmodium vivax* isolates and epidemiologic information on residents and migrants with parasitemia, Greece, 2009–2013.

Technical Appendix 2 Phylogenetic analyses of *Plasmodium vivax* isolates, Greece, 2009–2013.
