# Supplementary material for: Genetic Spatiotemporal Anatomy of Plasmodium vivax Malaria Episodes in Greece, 2009–2013
Source: Emerg Infect Dis. 2018 Mar;24(3):541–8. doi: 10.3201/eid2403.170605 (PMC5823331; doi:10.3201/eid2403.170605)
Supplement: Technical Appendix 2 — Phylogenetic analyses of Plasmodium vivax isolates, Greece, 2009–2013. [file 17-0605-Techapp-s2.pdf]

Article DOI: https://doi.org/10.3201/eid2403.170605

Genetic Spatiotemporal Anatomy of *Plasmodium vivax* Malaria Episodes in Greece, 2009–2013

Technical Appendix 2

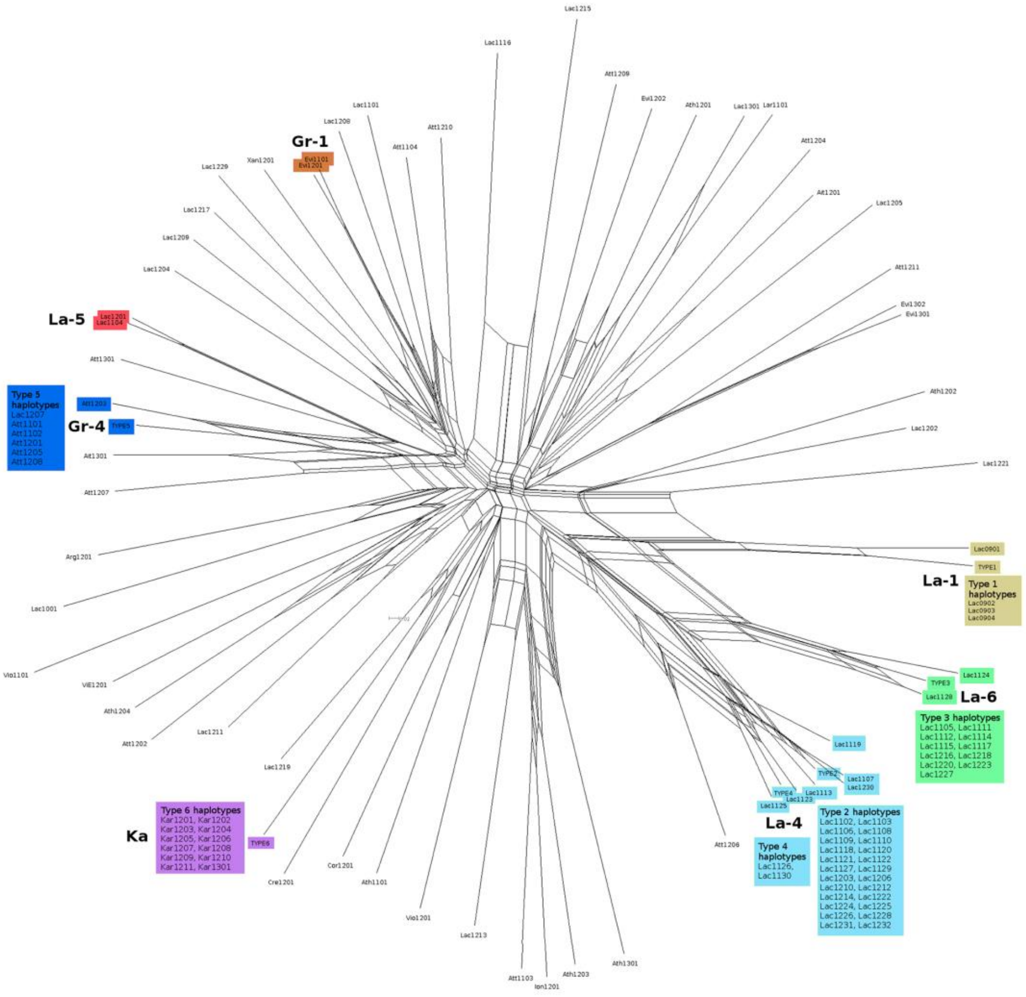

Technical Appendix Figure 1. Network phylogenetic analysis of the *Plasmodium vivax* isolates, Greece, 2009–2013. The haplotype families are highlighted with different colors. Detailed description of the haplotypes is provided in online Technical Appendix 1 Tables 1–3 (https://wwwnc.cdc.gov/EID/article/24/3/17-0605-Techapp1.xlsx).

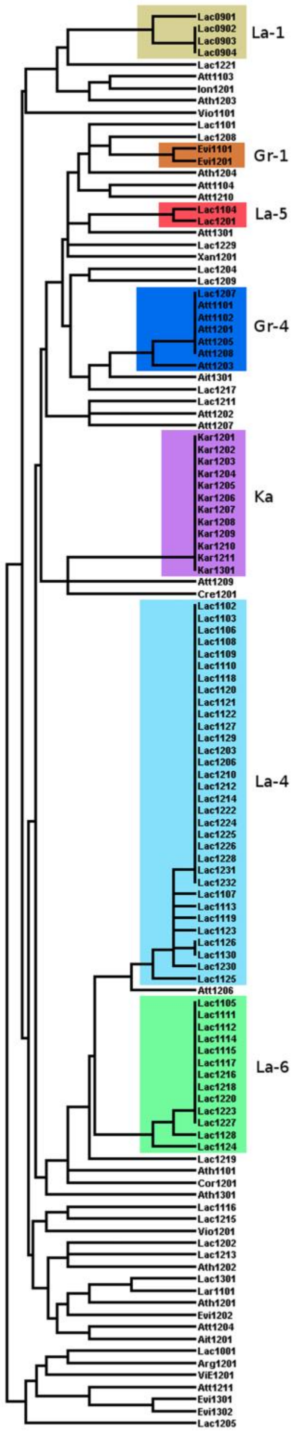

Technical Appendix Figure 2. Phylogenetic tree of the *Plasmodium vivax* isolates, Greece, 2009–2013. The haplotype families are highlighted with different colors. Detailed description of the haplotypes is provided in the online Technical Appendix 1 Tables 1–3 (https://wwwnc.cdc.gov/EID/article/24/3/17-0605-Techapp1.xlsx).
